# LC/MS- and GC/MS-based metabolomic profiling to determine changes in flavor quality and bioactive components of *Phlebopus portentosus* under low-temperature storage

**DOI:** 10.3389/fnut.2023.1168025

**Published:** 2023-06-29

**Authors:** Xiao-Bei Li, Chen-Menghui Hu, Cai-Hong Li, Guang-Yan Ji, Shun-Zhen Luo, Yang Cao, Kai-Ping Ji, Qi Tan, Da-Peng Bao, Jun-Jun Shang, Rui-Heng Yang

**Affiliations:** ^1^Shanghai Academy of Agricultural Sciences, Shanghai, China; ^2^Hongzhen Agricultural Science and Technology Co. Ltd., Jinghong, China

**Keywords:** LC/MS, GC/MS, *Phlebopus portentosus*, nutritional changes, geosmin

## Abstract

**Introduction:**

Low temperature is the most common method used to maintain the freshness of *Phlebopus portentosus* during long-distance transportation. However, there is no information regarding the nutritional changes that occur in P. portentosus preserved postharvest in low temperature.

**Methods:**

In this study, the changes in flavor quality and bioactive components in fruiting bodies stored at 4 °C for different storage periods were determined through LC/MS and GC/MS analyses. Sampling was performed at 0, 3, 5, 7, and 13 days storage.

**Results and Discussion:**

Based on the results, the metabolites present in caps and stipes were different at the same period and significantly different after 7 days of storage. A total of 583 and 500 different metabolites were detected in caps and stipes, respectively, and were mainly lipids and lipid-like molecules, organic acids and derivatives, organic oxygen compounds and others. Except for prenol lipids and nucleotides, the expression levels of most metabolites increased with longer storage time. In addition, geosmin was identified as the major contributor to earthy-musty odors, and the level of geosmin was increased when the storage time was short.

**Conclusion:**

The variations in these metabolites might cause changes in flavor quality and bioactive components in *P. portentosus*. Variations in these metabolites were thoroughly analyzed, and the results revealed how storage processes affect the postharvest quality of *P. portentosus* for the first time.

## Introduction

*Phlebopus portentosus* (Berk. and Broome) Boedijin was the first ectomycorrhizal fungus artificially cultivated in greenhouses (1–3). This mushroom is delectable and rich in nutrients, including polysaccharides, proteins, dietary fiber and mineral elements (4–6); as a result, the mushroom is widely considered a favorite ‘black bolete’ in China, Thailand and Vietnam. To meet market demands, technologies have been updated in companies and field inoculations with plants to cultivate this fungus (3). However, most production of this fungus (80%) occurs in Yunnan and Guizhou provinces, which are in southwest China and far from the top consumer cities, including Shanghai, Chengdu and Chongqing. Maintaining the freshness of *P. portentosus* during long-distance transportation is currently a challenge.

*Phlebopus portentosus* is highly perishable after harvest and cannot be stored at room temperature for more than 5 days; thus, it is difficult to market *P. portentosus* as a fresh mushroom when the product is transported over long distances. Postharvest preservation involves complex biological processes including deterioration (7, 8). Deterioration is the most serious problem in all horticulture goods, including fruits, vegetables and mushrooms, and is characterized by water loss, softening of texture, microbial attack, browning and so on (7). Based on genomic, transcriptomic and metabolomic datasets, physiobiochemical and cytological structural changes occur in *Agaricus bisporus* (J.E. Lange) Imbach, *Lentinula edodes* (Berk.) Pegler and some *Pleurotus* species in postharvest preservation (9–11). Modified atmosphere packaging (12, 13), pulsed light (14) and low temperature (15) gas have been used to extend the shelf life of mushrooms. Storing the mushrooms in low temperature is the most common method used to maintain the freshness of mushrooms (15) and is widely used for the long-distance transportation of *P. portentosus*. However, there are some disadvantages to this method, such as loss of nutrition, texture and flavor (16). Unfortunately, no information is available regarding the nutritional changes that occur in *P. portentosus* when the mushroom is preserved postharvest in low temperature. LC/MS- and GC/MS-based metabolomics are widely used to investigate the compositions of polysaccharides, amino acids and secondary metabolites in mushrooms under different developmental stages or treatments, e.g., *A. bisporus*, *L. edodes*, *Phallus rubrovolvatus* (M. Zang, D.G. Ji & X.X. Liu) Kreisel, *Hypsizygus marmoreus* (Peck) H.E. Bigelow and so on (17–22). Due to their high resolution, sensitivity and peak reproducibility, the methods are convenient for rapidly screening and identifying metabolites in *P. portentosus*.

In addition, fruiting bodies of *P. portentosus* exhibit an earthy-musty smell. Trans-1,10-dimethyl-trans-9-decalo (Geosmin, GSM), 2-methylisoborneo (MIB), 2-isopropyl-3-methoxypyrazine (IPMP), 2-isobutyl-3-methoxypyrazine (IBMP) and 2,3,6-trichloroanisole (TCA) are sources of this earthy-musty smell, and among the compounds, GSM and MIB are the most common (23). The same earthy and musty odor compounds have been found in drinking water, aquatic products, fruits and vegetables. Humans are extremely sensitive to these compounds and have very low thresholds, e.g., <10 ng/L for GSM (24, 25). Disagreeable tastes and odors in water or food has resulted in very large economic losses. These molecules are produced by many microorganisms, such as cyanobacteria, actinomycetes, protozoa, and fungi, during the development of products (23). The source determination of earthy-musty smell plays an important role in removal. However, there is no report on the earthy-musty smell of *P. portentosus*. The compounds that contribute to the earthy-musty smell remain unknown.

In this study, *P. portentosus* was preserved under low temperature for long periods. LC/MS and GC/MS were used to determine the changes in flavor quality and bioactive components at different storage times. This study provides information on the nutritional variations in *P. portentosus* after harvest.

## Materials and methods

### Artificial cultivation of *Phlebopus portentosus*

Pure culture strain P1 was collected from Xishuangbana, China, and isolated from a fruiting body, which was provided by Hongzhen Agricultural Science and Technology Co., Ltd. This fungus was incubated on modified PDA medium at 30°C in the dark. All cultivation methods, including preparing primary and secondary spawn and culturing the mushrooms, were performed according to previously published methods (26, 27). Sawdust, starch and soil were used as the cultivation substances. A 1100 mL culture bottle 8.5 cm in diameter and 14.0 cm in height was used. All bottles filled with substances were sterilized at 12°C for 2 h. The sterilized substances were cooled to room temperature and inoculated with the spawn of the fungus incubated at 28°C. Nearly 40 d were needed to obtain completely colonized hyphae. Afterward, the 2 cm casing soil covering the substrate hyphae in culture bottles was applied to induce the formation of primordia for 10 days of incubation. The fruit bodies were harvested in 5 days.

### Postharvest preservation under low temperature and sampling

To conduct postharvest analysis, the fruiting bodies were immediately transferred to a freezer and stored at 5°C. Sampling was performed at 0, 3, 5, 7, and 13 days (named G1, G3, G5, G7, G3, and B1, B3, B5, B7 and B13). Detailed information is listed in Supplementary Table S1. During sampling, a complete fruiting body was cut into two parts, cap and stipe, which were cut into 2-mm pieces and immediately frozen using liquid nitrogen. Then, the samples were stored at −80°C until analysis. A total of 6 duplicates were used at each period. Finally, a total of 60 samples, 30 caps and 30 stipes, were collected for metabolomic analysis.

### Metabolic sample preparation and LC–MS analysis

Sample preparations and LC–MS analysis were conducted according to previously published methods (28). Principal component analysis (PCA) and orthogonal partial least-squares-discriminant analysis (OPLS-DA) were performed to visualize the relationship among different samples using R packages. Hotelling’s T2 region, shown as an ellipse in the score plots of the models, defined the 95% confidence interval of the modeled variation. The R2X or R2Y and Q2 values were used to describe the quality of the models. The OPLS-DA models were also validated by a permutation analysis (200 times). The different metabolites were selected based on the variable influence on projection (VIP) values and *p* values obtained from the OPLS-DA model with a two-tailed Student’s t-test on the normalized peak areas. VIP values >1.5 and *p* values <0.05 were considered significant. Both datasets were analyzed on the free online Majorbio I-Sanger Cloud Platform.

### Determination of earthy-musty smell compounds using HS-SPME-GC–MS

The standards for GSM, MIB, IPMP, IBMP and TCA were obtained from Shanghai Anpel Scientific Instrument Corporation (Shanghai, China) and prepared at 0.25, 0.50, 1.0, 2.5, 5.0, 10.0 and 20.0 ng/g in matrix solution. A total of 1.0 g (± 0.05) fruiting body tissues were vortexed evenly with 4 g saturated NaCl solution. Earthy-musty smell compounds in the suspension were recovered using a 50/30 μM Carboxen/DVB/PDMS extraction fiber head (Merck Suplco). The extraction conditions were as follows: preextraction was performed at 60°C for 30 min and then extraction was performed using a fiber head for 30 min. The sample inlet was analyzed at 250°C for 5 min.

An Agilent 7890A-5975C gas chromatography–mass spectrometer (GC–MS) with a 60 m DB-WAX UI (250 μm inside diameter, 0.25 μm film thickness) capillary column (part No. 122-7062UI, Agilent J&W) was applied for analysis. The sample inlet temperature was 250°C. It was maintained at 40°C for 2 min, subsequently increased by 5°C/min to 220°C and held for an additional 3 min. The flow rate was 1.0 mL/min. Mass spectrum conditions were as follows: electron impact (EI) ion source, ionization voltage 70 eV, ion source 230°C, quadrupole 150°C, mass scan range 40–340 amu, running temperature 250°C.

## Results

### LC–MS analysis

The metabolites were identified through LC/MS to determine the chemical composition profile of *P. portentosus* under low-temperature storage at different times. A total of 1,135 metabolites were identified, and the numbers of positive and negative ion modes were 703 and 432, respectively. After normalization, 1,079 metabolites, including 667 positive and 412 negative ion modes, remained, which were used for later analysis.

**Figure 1:**
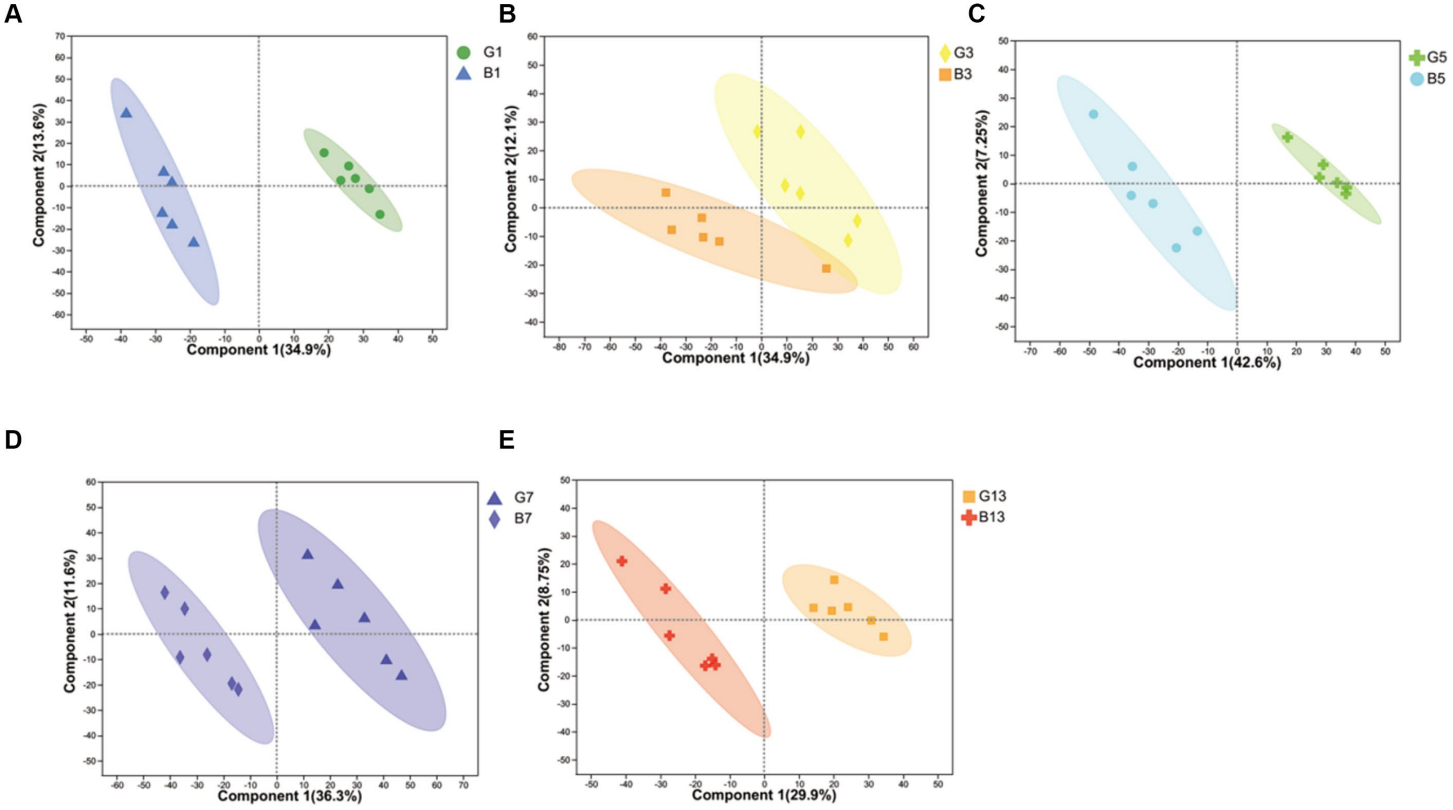
OPLS-DA plot-based metabolites identified in caps and stipes. **(A)** Samples collected at harvest. **(B)** Samples collected at Day 3. **(C)** Samples collected at Day 5. **(D)** Samples collected at Day 7. **(E)** Samples collected at Day 13.

In our study, cap and stipes were used to identify metabolites separately. The results obtained from the reduction in dimensionality of all datasets by OPLS-DA revealed that metabolite profiling in caps and stipes was different at every time point (Figure 1). For further analysis, caps and stipes were evaluated independently.

**Figure 2:**
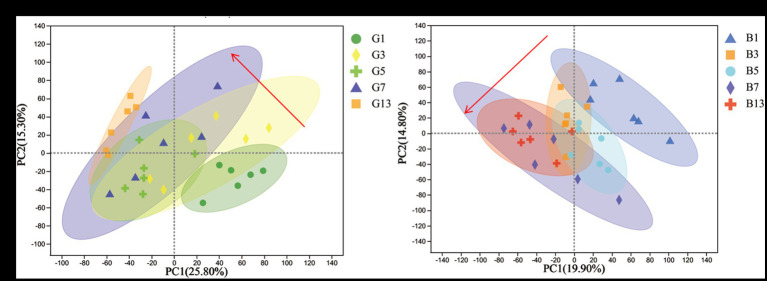
PCA plots of different samples stored under low temperature in caps **(A)** and stipes **(B)**.

To evaluate the effects of different storage times (Figure 2), the results of PCA revealed that metabolites produced by caps and stipes were different after 7 days of storage.

### Differential metabolite analysis at different storage times

For LC/MS analysis, a total of 583 (Supplementary File S1) and 500 (Supplementary File S2) different metabolites were detected among the 5 groups in caps and stipes, respectively.

**Figure 3:**
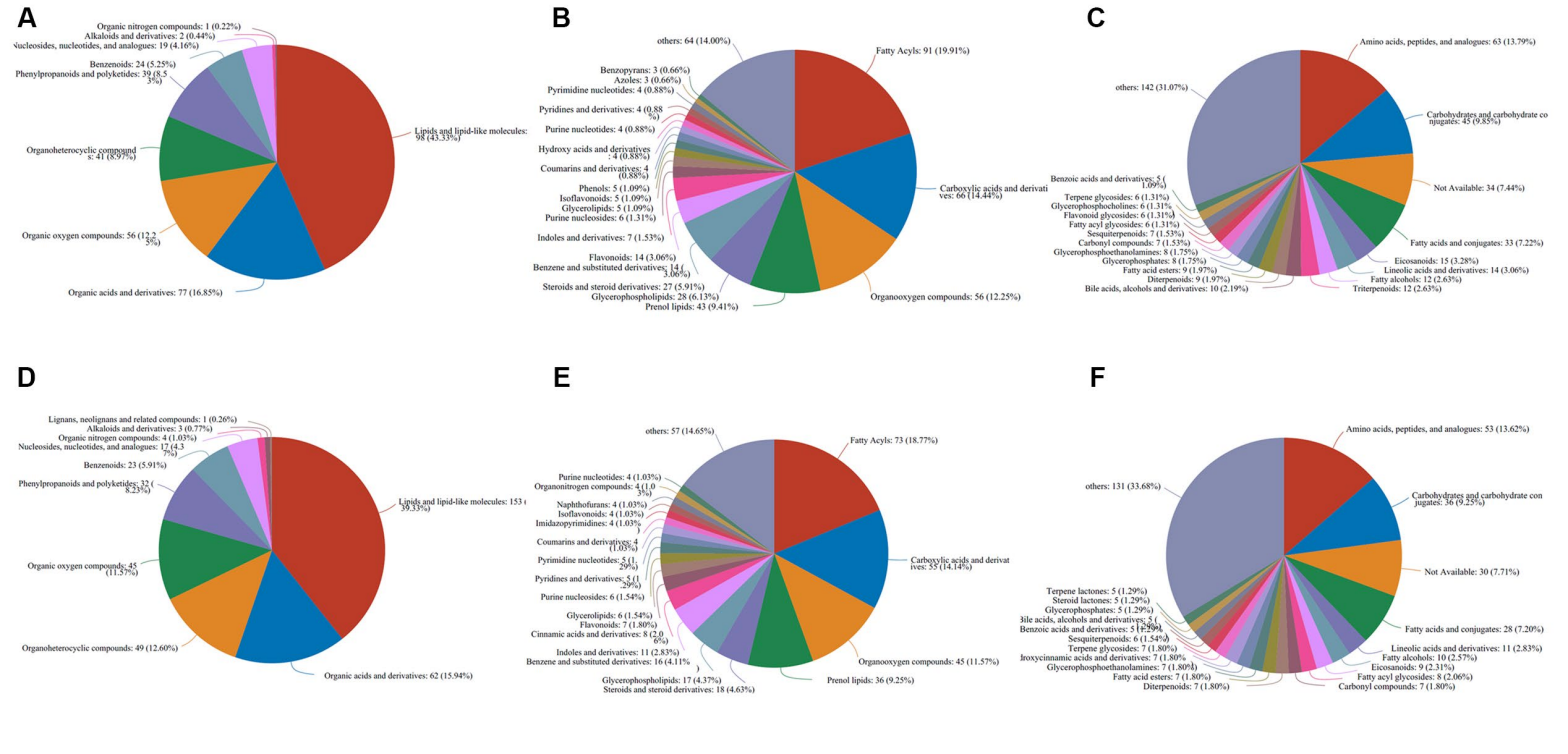
Classification and annotation of differential metabolites stored at different times in caps (583) and stipes (500). **(A)** Superclass in caps. **(B)** Class in caps. **(C)** Subclass in caps. **(D)** superclass in stipes. **(E)** Class in stipes. **(F)** Subclass in stipes.

The 583 metabolites in caps were mainly composed of 198 lipids and lipid-like molecules, 77 organic acids and derivatives, 56 organic oxygen compounds, 41 organoheterocyclic compounds, 39 phenylpropanoids and polyketides, 24 benzenoids, 19 nucleosides, nucleotides, and analogs, 2 alkaloids and derivatives and 1 organic nitrogen compound at the superclass level (Figure 3A). At the class and subclass levels (Figures 3B,C), a total of 64 and 107 compounds were annotated, respectively. The largest numbers of differential compounds at the class level were fatty acyls (91), carboxylic acids and derivatives (66), organooxygen compounds (56), prenol lipids (43) and glycerophospholipids (28). Amino acids, peptides, and analogs (63), carbohydrates and carbohydrate conjugates (45), fatty acids and conjugates (33), eicosanoids (15) and lineolic acids and derivatives (14) presented the top 5 subclasses.

The 500 different metabolites in stipes were annotated as 10 superclasses (Supplementary File S2 and Figure 3D), including lipids and lipid-like molecules (153), organic acids and derivatives (62), organoheterocyclic compounds (49), organic oxygen compounds (45), phenylpropanoids and polyketides (32), benzenoids (23), nucleosides and analogs (17), organic nitrogen compounds (4), alkaloids and derivatives (3) and lignans, neolignans and related compounds (1). A total of 62 and 104 classes and subclasses were annotated (Figures 3E,F). The top compound compositions were similar to the stipe (Figures 3D-F).

### Analysis of differential metabolites and expression according to storage time

Based on the differential metabolite analysis mentioned above, the differential metabolites belonging to lipids and organic acids were the most abundant. For comparative analysis, both kinds of metabolites were the main focus. Some phenylpropanoids and polyketides, nucleotides, and their derivatives are also related to flavor and quality in mushrooms.

### Lipids and lipid-like molecules

**Figure 4:**
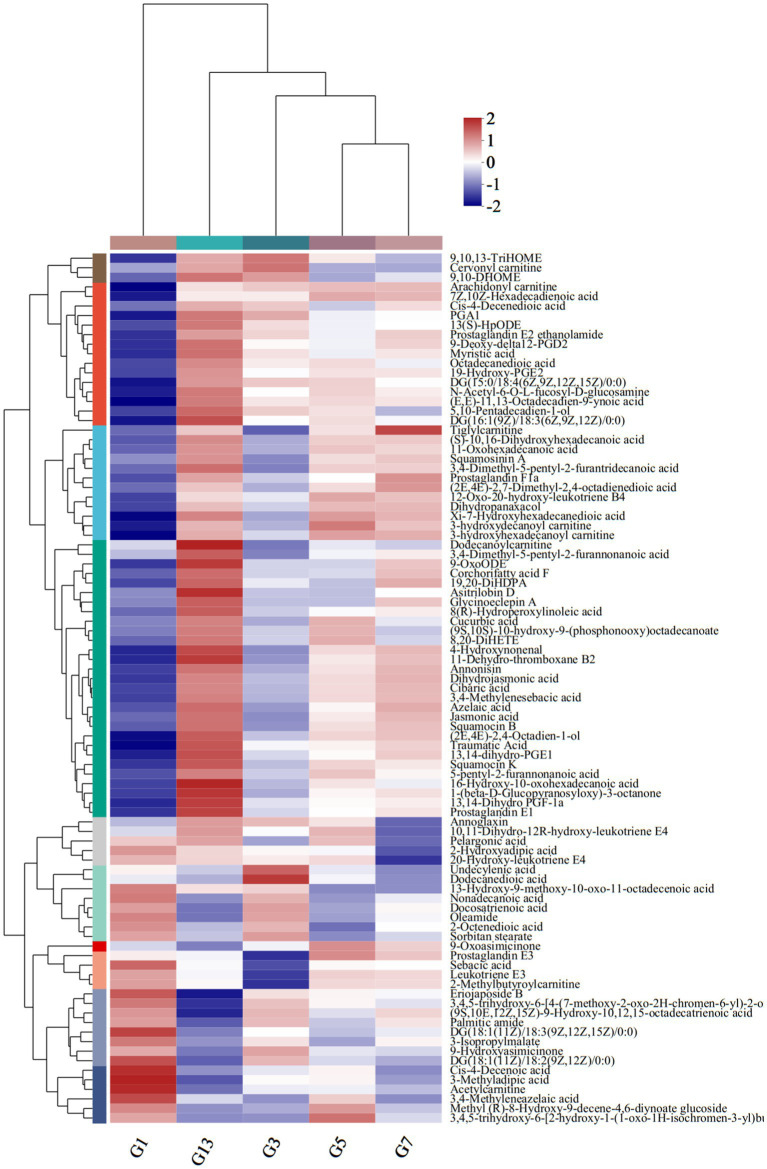
Heatmap clustering of fatty acyl metabolites in caps under low-temperature storage.

**Figure 5:**
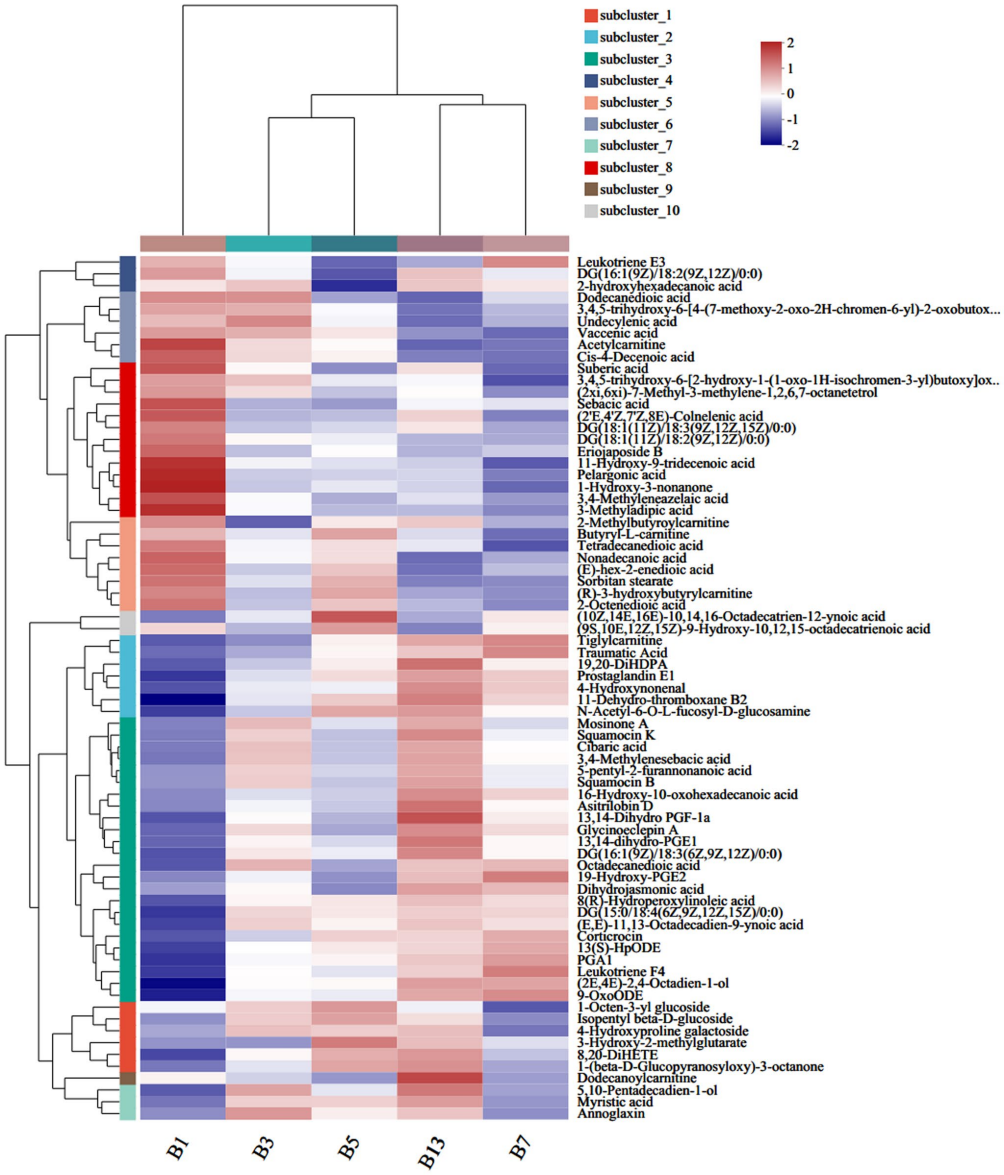
Heatmap clustering of fatty acyl metabolites in stipes under low-temperature storage.

Among the 583 different metabolites in caps, up to 198 lipids and lipid-like molecules, including 91 fatty acyls, 43 prenol lipids, 28 glycerophospholipids, 27 steroids and steroid derivatives, 5 glycerolipids, 3 sphingolipids and 1 endocannabinoid, were detected (Supplementary File S1). Of the 91 fatty acyls, the majority significantly increased with storage time. 3-Methyladipic acid, acetylcarnitine and cis-4-decenoic acid were the most abundant in G1. The highest levels of dodecanoylcarnitine, 16-hydroxy-10-oxohexadecanoic acid and asitrilobin D were found in G13 (Figure 4). In the stipes (Figure 5 and Supplementary File S2), lipids and lipid-like molecules were the most abundant different compounds, which decreased from harvesting to 3 days. After Day 3, the number of different metabolites increased. For example, 8 fatty acyl compounds were higher in B1 than in B3, while 17 fatty acyl compounds were higher in B3 than in B1. Among these pelargonic acids, 1-hydroxy-3-nonanone, eriojaposide B, 11-hydroxy-9-tridecenoic acid and sebacic acid were more abundant in B1 than in B3. Annoglaxin, 9-oxoode, (2E,4E)-2,4-octadien-1-ol, 11-dehydro-thromboxane B2 and 5,10-pentadecadien-1-ol showed higher levels in B3 than in B1. In Group B5 vs. B3, the levels of 2-methylbutyroylcarnitine, 3-hydroxy-2-methylglutarate, N-acetyl-6-O-L-fucosyl-D-glucosamine, 11-dehydro-thromboxane B2, (10Z,14E,16E)-10,14,16-octadecatrien-12-ynoic acid and (9S,10E,12Z,15Z)-9-hydroxy-10,12,15-octadecatrienoic acid varied the most. Compared with that in B5 (isopentyl beta-D-glucoside, 1-octen-3-yl glucoside, butyryl-L-carnitine, tetradecanedioic acid and 3-hydroxy-2-methylglutarate expressed higher levels), leukotriene E3, 19-hydroxy-PGE2 and leukotriene F4 were more abundant in B7. In the B13 vs. B7 group, only 1 compound (leukotriene E3) was higher in B7 than in B13, and 13 compounds were higher in B13 than in B7, including dodecanoylcarnitine, 5,10-pentadecadien-1-ol13,14-dihydro, GF-1a1-(beta-D-glucopyranosyloxy)-3-octanone, and 11-hydroxy-9-tridecenoic acid.

**Figure 6:**
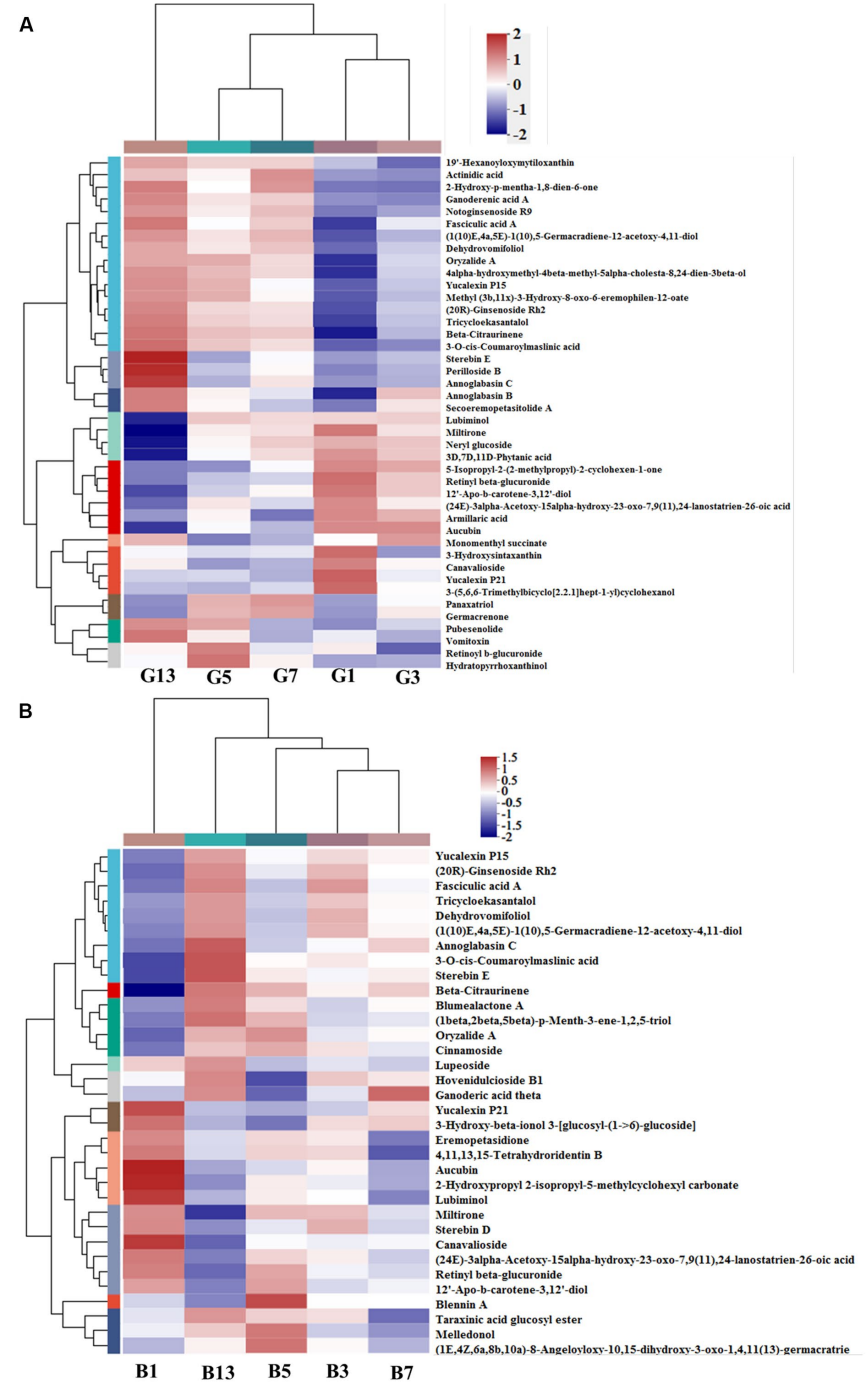
Heatmap clustering of the different prenol lipid metabolites in caps **(A)** and stipes **(B)** under low-temperature storage.

In the prenol lipid class, a total of 42 different terpenes were identified in caps in the five groups, including 12 triterpenoids, 9 diterpenoids, 7 sesquiterpenoids, 6 terpene glycosides, 5 monoterpenoids and 3 terpene lactones (Supplementary File S1). These terpene compounds are important secondary metabolites in mushrooms and play essential roles in the metabolism of organisms. Some diterpenoids (miltirone, 3D,7D,11D-phytanic acid, 12′-Apo-b-carotene-3,12′-diol, (24E)-3alpha-acetoxy-15alpha-hydroxy-23-oxo-7,9(11),24-lanostatrien-26-oic acid and Yucalexin P21), 4 triterpene glycosides and 3 monoterpenoids showed high levels in fresh mushrooms (Day 1). However, only 1 triterpenoid (3-hydroxysintaxanthin) and sesquiterpenoids (armillaric acid) were abundant in G1 (Figure 6A). With longer storage, the levels of triterpenoid and sesquiterpenoid compounds increased gradually. Diterpenoids and terpene glycosides compounds were the most abundant in sample G13, including stereobin E, perilloside B and annoglabasin C (Figure 6A). In stipes (Figure 6B), the variations in terpene compounds were similar to those in caps, with higher categories and abundances of related compounds with longer storage times. The number of triterpenoids was higher in B13 than in B1. 3-O-cis-Coumaroyl maslinic acid and beta-citraurinene were the most abundant in B11. Compared with B1, the levels of annoglabasin C, diterpenoids, and 3-o-cis-coumaroyl maslinic acid were higher than those in the other samples. 12′-Apo-b-carotene-3,12′-diol, aucubin, canavalioside, lubiminol, miltirone, retinyl beta-glucuronide and Yucalexin P21 were more abundant in the Day 1 samples (caps and stipes) than in the other samples.

In stipes and caps, the number of different compounds belonging to steroids and glycerophospholipids was higher with longer storage times (Supplementary Files S1, S2).

### Organic acids and derivatives

**Figure 7:**
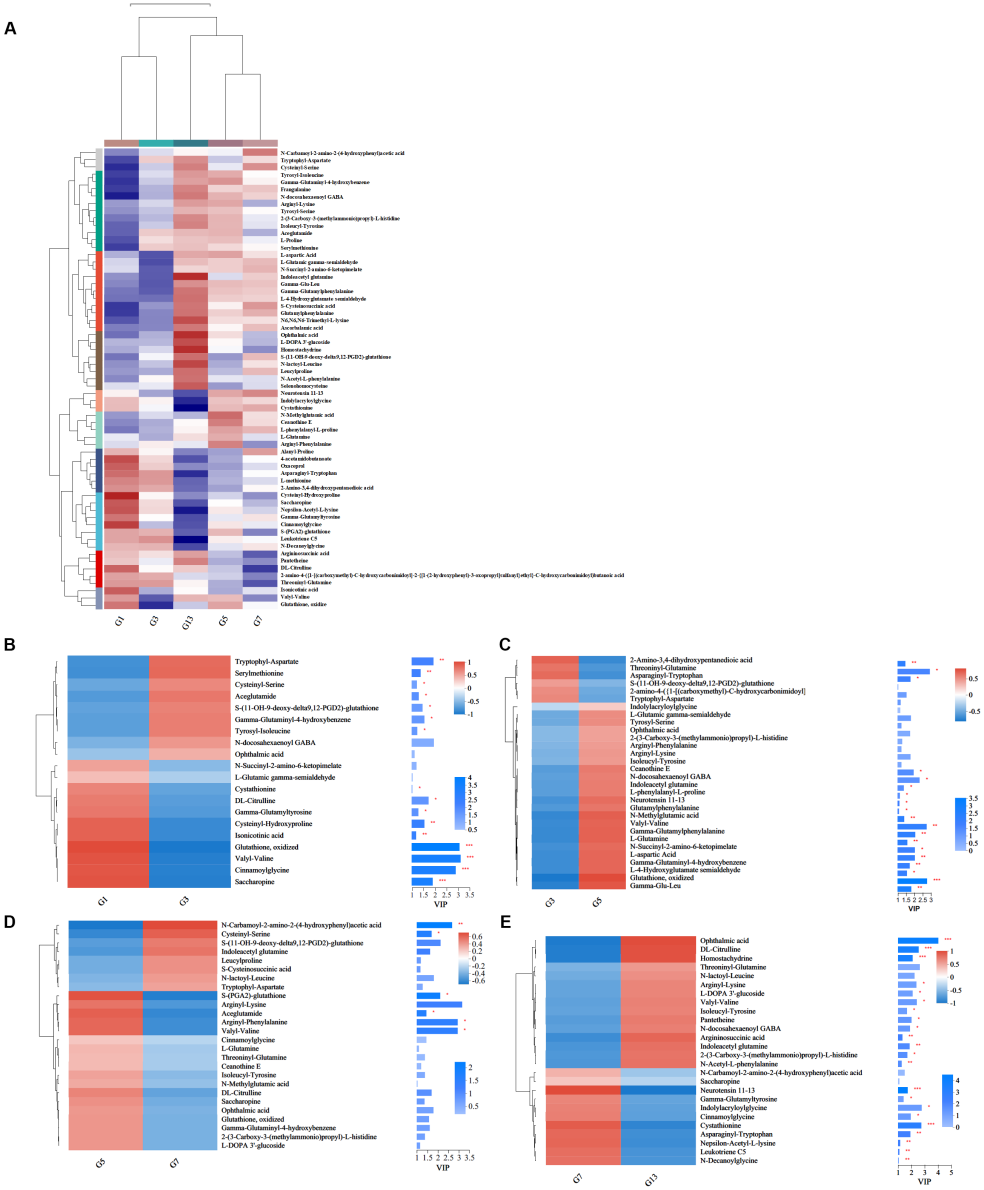
Heatmap clustering and VIP of the different amino acid metabolites in caps under low-temperature storage. **(A)** Heatmap analysis. **(B)** G3 vs. G1. **(C)** G5 vs. G3. **(D)** G7 vs. G5. **(E)** G13 vs. G7.

**Figure 8:**
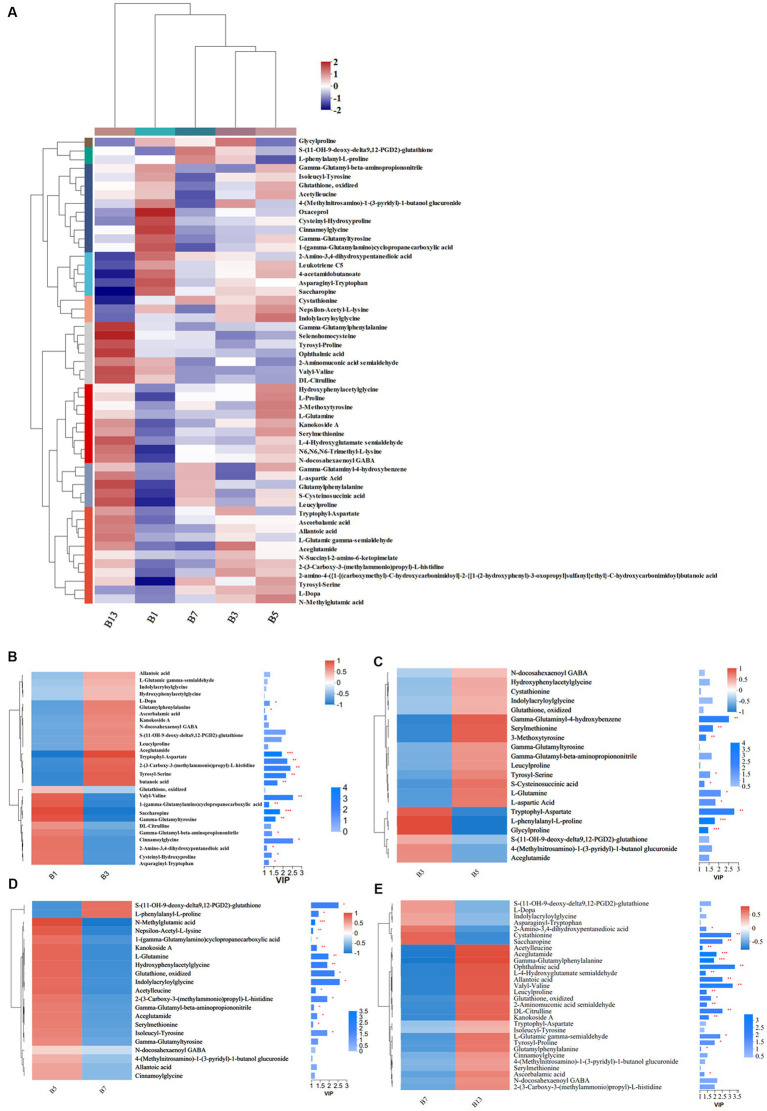
Heatmap clustering and VIP of the different amino acid metabolites in stipes under low-temperature storage. **(A)** Heatmap analysis. **(B)** B3 vs. B1. **(C)** B5 vs. B3. **(D)** B7 vs. B5. **(E)** B13 vs. B7.

Storage time also influenced some metabolites of organic acids and derivatives related to flavor and quality. Based on the metabolic comparisons in caps (Supplementary File S1), the number of different metabolites was 77, and these metabolites were assigned into 8 different subclasses, of which 63 metabolites were from the class of amino acids, peptides, and analogs (accounting for 85.71% of total different organic acids and derivatives). The number of different amino acids, peptides, and analogs decreased after harvest. However, the amino acid profiles varied, and the categories of different amino acid compounds increased under long-term storage (Figure 7A). The VIP scores were obtained based on the OPLS-DA models and were used to determine the influence intensity and most discriminating amino acid compounds between different groups. Glutathione, valyl-valine, cinnamoylglycine and saccharopine were the most abundant in G1, and tryptophyl-aspartate, serylmethionine, S-(11-OH-9-deoxy-delta9,12-PGD2)-glutathione and gamma-glutaminyl-4-hydroxybenzene were the most significant compounds in G3 (Figure 7B). Only the levels of 2-amino-3,4-dihydroxypentanedioic acid, threoninyl-glutamine and asparaginyl-tryptophan were significantly higher in G3 than in G5 (Figure 7C). In G5, the contents of glutathione, gamma-glutamyl, N-methylglutamic acid and valyl-valine increased after storage for more than 3 days (Figure 7C). The expression levels of N-carbamoyl-2-amino-2-(4-hydroxyphenyl) acetic acid and cysteinyl-serine on Day 7 were higher (Figure 7D). However, S-(PGA2)-glutathione, aceglutamide, and arginyl-phenylalanine decreased. For long-term storage, more amino acids, peptides, and analogs varied significantly, reaching 13 compounds in G13, e.g., ophthalmic acid, DL-citrulline and homostachydrine. The expression of some other compounds (9) was also low, especially neurotensin 11–13 and cystathionine (Figure 7E). In the stipes, 53 different amino acid compounds were detected (Supplementary File S2). During the 13 days of storage, the contents of amino acid compounds varied significantly and decreased with longer storage time (Figure 8A). Valyl-valine, 1-(gamma-glutamylamino)cyclopropanecarboxylic acid, saccharopine and gamma-glutamyltyrosin showed higher levels in stipes of fresh mushroom fruiting bodies in B1 than in B3 (Figure 8B). Aceglutamide, tryptophyl-aspartate, 2-(3-carboxy-3-(methylammonio)propyl)-L-histidine, tyrosyl-serine, and 2-amino-4-({2-[(1-carboxy-1-hydroxy-2-methylpropan-2-yl)sulfanyl]-1-[(carboxymethyl)-C-hydroxycarbonimidoyl]ethyl}-C-hydroxycarbonimidoyl)butanoic acid were the biomarkers in the samples collected at Day 3 (B3). Gamma-glutamyl-4-hydroxybenzene, serylmethionine, and 3-methoxytyrosine accounted for the most different amino acids in B5 (Figure 8C). However, most amino acid compounds decreased in B7, especially L-glutamine, hydroxyphenylacetylglycine, glutathione, oxidized and indolylacryloylglycine. S-(11-OH-9-deoxy-delta9,12-PGD2)-glutathione and L-phenylalanyl-L-proline showed high expression levels in B7 (Figure 8D). B13 contained high contents of more amino acids, similar to other compounds covered in this study (16). Among these metabolites, storage time promoted the expression of acetylleucine, aceglutamide, gamma-glutamylphenylalanine, ophthalmic acid and so on (Figure 8E).

### Phenylpropanoids and polyketides, nucleotides, and their derivatives

In the superclass phenylpropanoids and polyketides (Supplementary Files S1, S2), 19 out of 39 compounds were identified as isoflavonoids (5) and flavonoids (14) in caps. The variations in the five groups revealed that flavonoid glycosides were abundant in G1, which decreased after 3 days of storage (Supplementary File S1). Some other flavonoids exhibited significant changes, and the number of these compounds decreased (Supplementary File S1). These compounds in stipes showed the same variation trends as those in caps, and flavonoid glycosides were also the most attractive in B1 and decreased later. Flavonoids replaced flavonoid glycosides as the most abundant compounds (Supplementary File S2).

**Figure 9:**
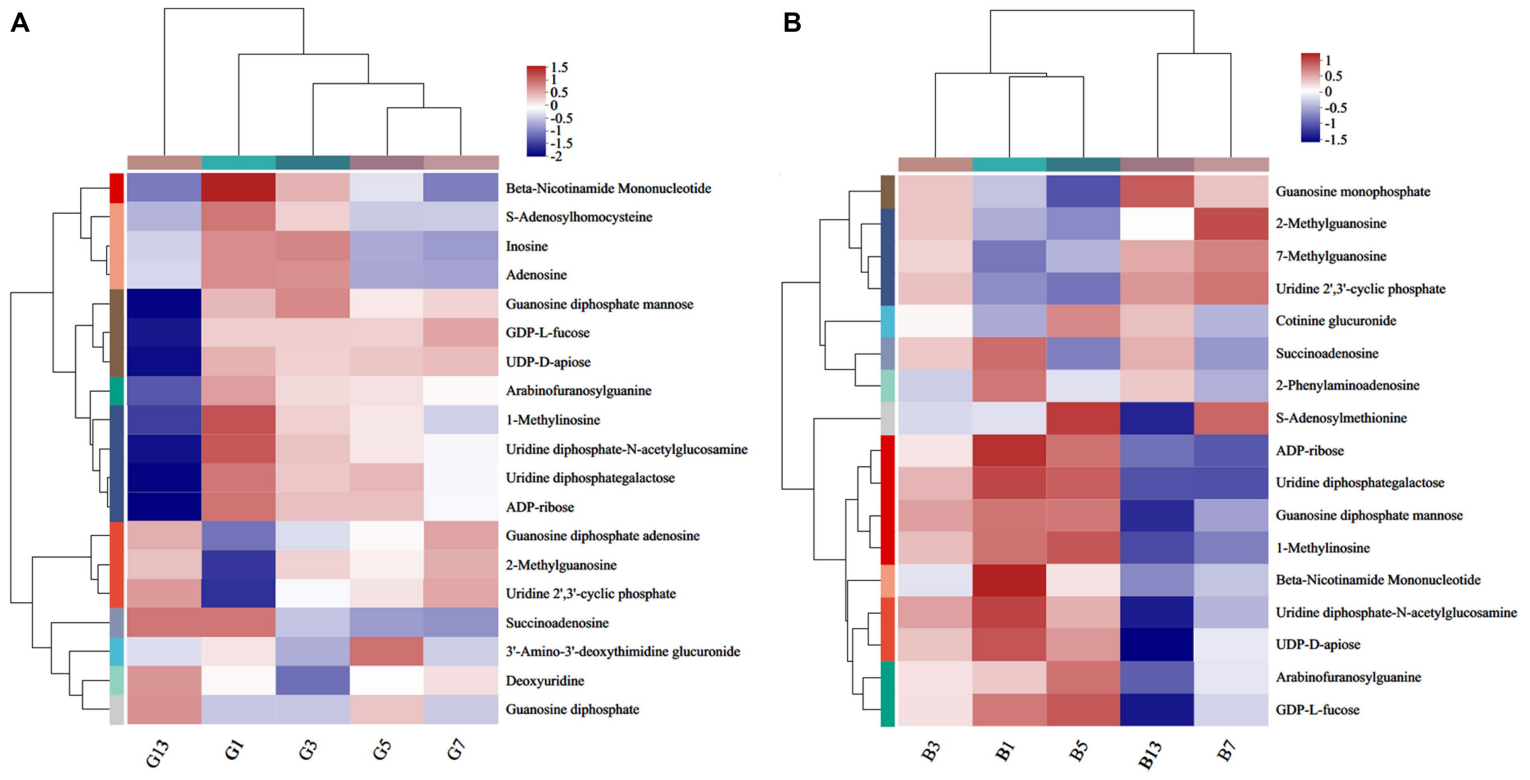
Heatmap clustering of the different nucleotide metabolites in caps **(A)** and stipes **(B)** under low-temperature storage.

A total of 19 (Figure 9A) and 17 (Figure 9B) different nucleotides were detected in the caps and stipes, respectively, across all groups. Overall, a lower expression of metabolites was observed during the storage processes. For example, beta-nicotinamide mononucleotide, 1-methylinosine, uridine diphosphate-N-acetylglucosamine, and uridine diphosphategalactose were abundant in G1 and were nearly absent in G13 (Figure 9A). Beta-nicotinamide mononucleotide and ADP-ribose slowly lowered the expression, and guanosine monophosphate, 7-methylguanosine and uridine 2′,3′-cyclic phosphate showed higher levels in stipes (Figure 9B).

### Determination of earthy-musty smell compounds using HS-SPME-GC–MS

Quantitative and qualitative ion information of the five standards is shown in Table 1. Four grams of saturated NaCl solution and 100 μL of mixed standard solution (acetone solution, adding concentration of 2.5–200 μ G/L) were used as the matrix standard solution after being placed at room temperature for 1 day. The standard curve was drawn with the concentration of the standard solution as the horizontal coordinate (x) and the abundance value of the corresponding peak area as the vertical coordinate (y). After the background value of the matrix sample was deducted, the regression equation and the correlation coefficients were calculated, as listed in Table 2. The calibration curves showed good linearity for all compounds, and the correlation coefficients ranged from 0.993 to 0.998.

**Table 1:** MS information for the five standards.

Compound	Retention time (min)	Mass fragments (m/z)
IPMP	10.274	137*, 152, 124
IBMP	11.363	124*, 151, 94
2MIB	12.395	95*, 108, 135
GSM	16.197	112*, 125, 97
TCA	17.899	212*, 197, 167

* Quantifier ion.

**Table 2:** Linear ranges, regression equations, correlation coefficients, recovery, limit of detection (LOD) and limit of quantitation (LOQ) (*n* = 6).

Compounds	Regression equations ^a^	Correlation coefficient (R^2^ ^b^)	1 μg/kg ^c^		2.5 μg/kg		10 μg/kg		LOD ^d^	LOQ ^e^
			Recovery/%	RSD/%	Recovery/%	RSD/%	Recovery/%	RSD/%	(μg/kg)	(μg/kg)
IPMP	*y* = 19798x-s1324	0.9959	96.83	19.24	106.17	15.08	100.41	13.85	0.05	0.16
IBMP	*y* = 49201x-16269	0.9932	103.78	11.55	103.63	6.04	92.01	12.95	0.02	0.08
2MIB	*y* = 18217x-3576	0.9982	105.13	19.11	103.52	5.36	106.28	6.09	0.14	0.46
GSM	*y* = 27751x-2917	0.9956	104.13	12.41	100.51	7.47	97.34	14.07	0.06	0.21
TCA	*y* = 11988x-2,495	0.9989	108.06	9.02	113.08	7.3	109.87	15.43	0.01	0.03

^a^ Expressed as the mass ratio (m:m) of the earthy substance to the sample.

^b^ The regression equation was calculated after deducting the background value of the matrix sample.

^c^ Spiked concentration.

^d^ Based on S/N = 3.

^e^ Based on S/N = 10.

The accuracy of the method was investigated by adding three levels of mixed standard solution (1, 2.5, 10 μg/kg) into the matrix solution, and each concentration was repeated 6 times. The limit of detection (LOD) was calculated by 3 times the signal-to-noise ratio (S/N = 3), and the limit of quantitation (LOQ) was calculated by S/N = 10. As shown in Table 2, the LOD of the 5 compounds in SIM mode was within 0.01–0.14 μg/kg, while the LOQ ranged from 0.03 to 0.46 μg/kg. Recoveries of 5 compounds in *P. portentosus* ranged from 92.01 to 113.08% at 1, 2.5 and 10 μg/kg spiked levels, and the relative standard deviations (RSDs) ranged from 5.36 to 19.24%. The developed analytical method was suitable for the determination of ground compounds in *P. portentosus*.

**Figure 10:**
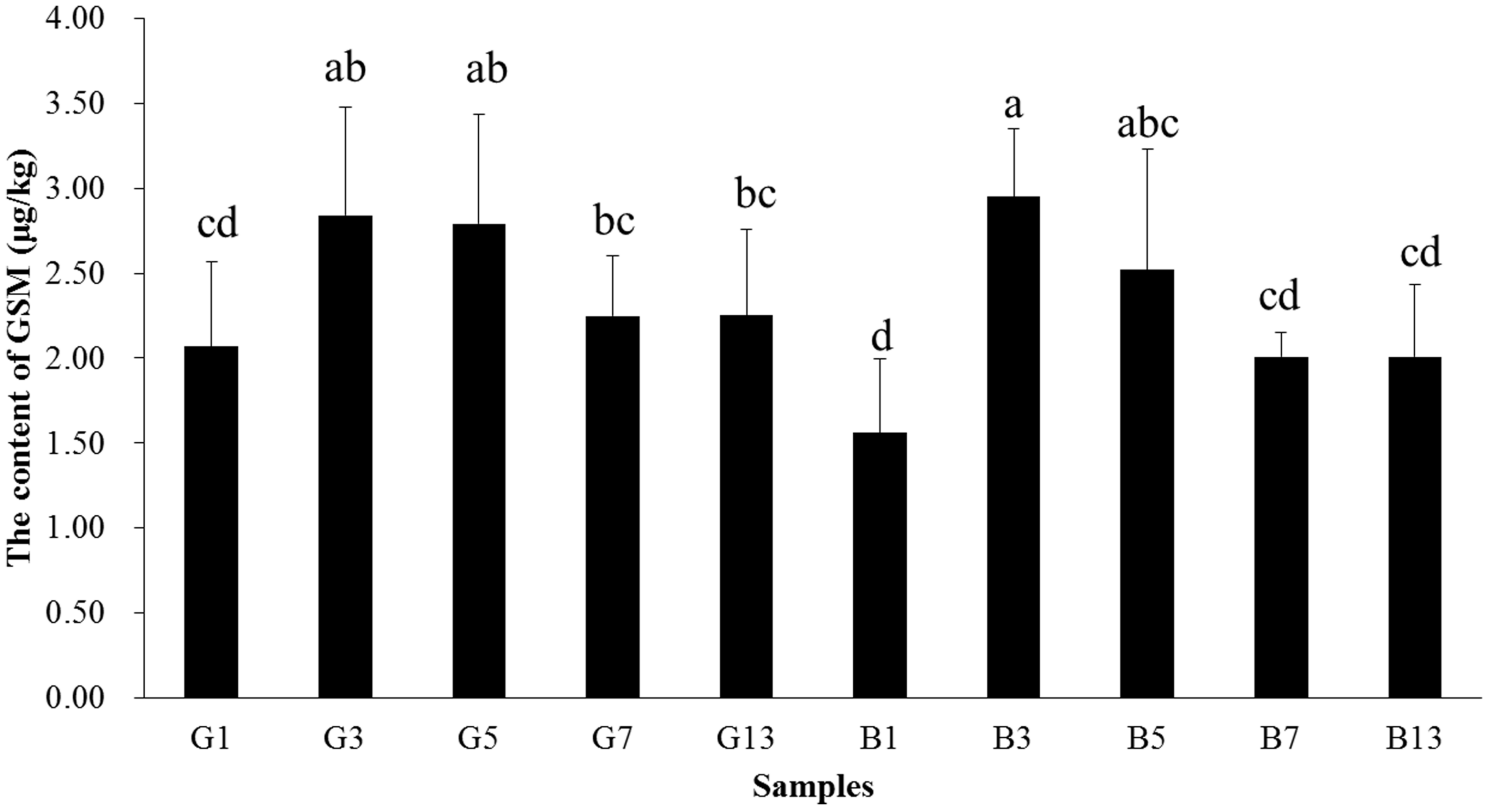
Concentrations of GSM in the caps and stipes of different samples stored at low temperature. Different lowercase letters reveal significant differences.

The content of GSM in fruiting bodies increased before storage time < 5 days, and the content decreased afterward. In samples B13 and G13, the GSM content was 2.25 and 2.01 μg/kg, respectively, which was paralleled with B1 and G1. In fresh fruiting bodies, the GSM content in the cap was higher than that in the stipes. However, as the storage time increased, no differences were observed between caps and stipes (Figure 10).

## Discussion

In this study, LC/MS and GC/MS analyses were used to determine the changes in flavor quality and bioactive components in *P. portentosus*. The results revealed that after 7 days of storage, the metabolomic profiles were different from those of fresh samples, especially for lipids and organic acids. In addition, earthy-musty smell compounds were determined using HS-SPME-GC–MS, and these compounds varied during the storage process.

In this study, metabolomics in caps and stipes were different, which were separated based on reducing the dimensionality analysis of OPLS-DA. The result was in agreement with the results obtained from *Hypsizygus marmoreus* and *Agaricus bisporus*, showing spatial metabolomic variations between different parts (21, 29, 30). In some other ectomycorrhizal fungi (31), e.g., *Russula cyanoxantha*, *Amanita rubescens*, *Suillus granulatus*, and *Boletus edulis*, some phenolic, organic acid and alkaloid compositions were different in caps and stipes in different species. Some transcriptomic research has also revealed the specificity of spatial expression in different parts (30). In this study, more different metabolites were found in the cap than stipes; high levels of amino acids, fatty acids, and N-acetylglucosamine were found in the caps, and more β-glucans, malic acid, and fructose were found in the stipes. All the differences may be due to the different functions of different parts of mushrooms. Compared with stipes, cap regions play important roles in reproductive functions, including spore production, development, and dispersion; thus, cap regions require more energy, amino acids and nucleotides (32). Stipes are mainly responsible for the transportation of water and solutes and supported the cap (33).

After harvest, lipids and organic acids tend to increase over long-term storage. In *P. tuoliensis*, the levels of some fatty acids increased (10). The expression of some enzymes involved in many pathways was higher after harvest, e.g., carbohydrate metabolism, which might catalyze the production of more metabolites and biomarkers for deterioration (10, 11). In addition, storage under low temperature resulted in a growth and maturation process; thus, more energy and metabolites were consumed for development (21, 34). Some lipids can regulate cell physiology, exert anti-inflammatory effects, reduce cholesterol levels and participate in flavoring compound synthesis (35). However, lipids and proteins decreased after harvest, which differs from our results, and the reasons need to be further studied. Terpene glycosides, sesquiterpenoids and diterpenoid compounds are important secondary metabolites for cell growth and metabolism (36) and possess antibacterial and antitumor activities. The increase in these compounds revealed that the fruiting bodies might resist attack by microorganisms. The amino acid profile of potatoes and *P. tuoliensis* revealed that some amino acids increased when stored at 0°C (37). Amino acids are essential compounds for mushroom growth and have many nutritional and physiological functions, e.g., converting soluble proteins to free amino acids or the synthesis of other amino acids (38–40). In our study, 63 and 53 different amino acid compounds were detected across all samples. Glutathione, which is abundant in G1, functions as an antioxidant in nearly all organisms and plays important roles in detoxification, posttranslational regulation and immune regulation (41). Ophthalmic acid, which is a biomarker of oxidative stress, was the most different amino acid in G13 (42). The variations in amino acids with different tastes caused the flavor quality in *P. portentosus* to change.

The compounds belonging to phenylpropanoids, polyketides and nucleosides decreased after storage, which might impact the quality and flavor of mushrooms. It has been acknowledged that nucleotides participate in almost all biological processes and are umami-taste active components in mushrooms (43, 44). Beta-nicotinamide mononucleotide and uridine diphosphate-N-acetylglucosamine exhibited higher levels in fresh fruiting bodies and decreased slowly. All these metabolites function as flavor and taste substances, and changes in these metabolites may lead to changes in taste and flavor (17).

In *P. portentosus*, the earthy-musty smell compound mainly resulted from geosmin. The common methods used to determine earthy-musty smell compounds reported in the literature usually require a preprepared step followed by gas chromatography–mass spectrometry (45, 46). Reports have suggested that headspace SPME (HS-SPME) is effective for collecting volatile organic compounds from samples with no solvent during extraction and is among the most popular techniques used for pretreating and enriching odorants (47–51). Geosmin is produced by cyanobacteria, actinomyces, fungi, and blue–green algae. It was reported that *Botrytis cinerea* and *Penicillium* in fungi could synthetize this compound (23, 52, 53). However, no reports involved earthy-musty smell-related compounds in mushrooms except for a report on 1-octen-3-ol. This study was the first research involving geosmin in *P. portentosus*. However, the origins of geosmin remain unknown, and other experiments need to be conducted. The threshold of geosmin is very low for humans (less than 10 ng/L); thus, the compound is recognizable at very low concentrations (24, 25). The concentrations of geosmin in *P. portentosus* ranged from 13.56 to 2.95 μg/kg, which is much higher than the threshold. Therefore, geosmin threatens the development of some industries and leads to economic losses. Additionally, when the samples were stored at a low temperature, the level of geosmin increased. However, after 7 days, the concentration of geosmin decreased. The production of geosmin was not decreased by low-temperature storage. Determining the compound that contributes to earthy-musty smell was the first step, and removing GSM molecules is more important for *P. portentosus* production.

## Conclusion

In this study, metabolomic profiling of *P. portentosus* stored under low temperature was determined using LC/MS and GC/MS analysis. The results revealed that long-term storage changed the metabolite compositions in fruiting bodies, flavor quality and bioactive functions. In addition, geosmin was determined to be the major contributor to earthy-musty smells. However, long storage time could not decrease this compound. This study provides information on the nutritional and flavorful variations of *P. portentosus* after harvest and storage. To maintain the freshness of *P. portentosus* and prolong its shelf life, new preservation methods need to be designed.

## Data availability statement

The original contributions presented in the study are included in the article/Supplementary material, further inquiries can be directed to the corresponding authors.

## Author contributions

D-PB, K-PJ, J-JS, R-HY, and QT contributed to the conception and design of the study. X-BL, C-MH, and R-HY conducted the experiments. C-HL, G-YJ, S-ZL, YC, and G-YJ prepared the samples. J-JS, R-HY, and K-PJ performed the analysis and wrote the manuscript draft. All authors approved the final version of the manuscript.

## Funding

This work was supported by the Shanghai Action Plan for Scientific and Technological Innovation (20310741800) and the National Natural Science Foundation of China (32072644).

## Conflict of interest

K-PJ, YC, S-ZL, and G-YJ are employed by Hongzhen Agricultural Science and Technology Co. Ltd.

The remaining authors declare that the research was conducted in the absence of any commercial or financial relationships that could be construed as a potential conflict of interest.

## Publisher’s note

All claims expressed in this article are solely those of the authors and do not necessarily represent those of their affiliated organizations, or those of the publisher, the editors and the reviewers. Any product that may be evaluated in this article, or claim that may be made by its manufacturer, is not guaranteed or endorsed by the publisher.
