# Supplementary material for: LC/MS- and GC/MS-based metabolomic profiling to determine changes in flavor quality and bioactive components of Phlebopus portentosus under low-temperature storage
Source: Front Nutr. 2023 Jun 29;10:1168025. doi: 10.3389/fnut.2023.1168025 (PMC10349180; doi:10.3389/fnut.2023.1168025)
Supplement: Supplementary file 1 [file Table_1.docx]

**TABLE S1.** The detailed information of samples

| Sample name | Sampling position | Sample time (day) | Repetitions |
| --- | --- | --- | --- |
| G1 | Gap | 0 | 6 |
| G3 | Gap | 3 | 6 |
| G5 | Gap | 5 | 6 |
| G7 | Gap | 7 | 6 |
| G13 | Gap | 13 | 6 |
| B1 | Stipe | 0 | 6 |
| B3 | Stipe | 3 | 6 |
| B5 | Stipe | 5 | 6 |
| B7 | Stipe | 7 | 6 |
| B13 | Stipe | 13 | 6 |
